# Inflammatory Dietary Pattern, *IL-17F* Genetic Variant, and the Risk of Colorectal Cancer

**DOI:** 10.3390/nu10060724

**Published:** 2018-06-05

**Authors:** Young Ae Cho, Jeonghee Lee, Jae Hwan Oh, Hee Jin Chang, Dae Kyung Sohn, Aesun Shin, Jeongseon Kim

**Affiliations:** 1 Department of Cancer Biomedical Science, Graduate School of Cancer Science and Policy, National Cancer Center, Goyang 10408, Gyeonggi-do, Korea; youngcho914@gmail.com (Y.A.C.); jeonghee@ncc.re.kr (J.L.); 2 Center for Colorectal Cancer, National Cancer Center Hospital, National Cancer Center, Goyang 10408, Gyeonggi-do, Korea; jayoh@ncc.re.kr (J.H.O.); heejincmd@ncc.re.kr (H.J.C.); gsgsbal@ncc.re.kr (D.K.S.); 3 Department of Preventive Medicine, Seoul National University College of Medicine, Seoul 03080, Korea; shinaesun@snu.ac.kr

**Keywords:** colorectal cancer, inflammation, C-reactive protein, dietary pattern, *IL-17F* polymorphism

## Abstract

A proinflammatory diet may increase the risk of colorectal cancer, but its role may differ according to individuals’ genetic variants. We aimed to examine whether a specific dietary pattern reflecting inflammation was associated with a risk of colorectal cancer and whether *IL-17F* genetic variant altered this association. In a study of 695 colorectal cancer cases and 1846 controls, we derived a reduced rank regression dietary pattern using 32 food groups as predictors and the plasma C-reactive protein (CRP) concentration as the response. High CRP levels were associated with a high risk of colorectal cancer (OR (95% CI) = 3.58 (2.65–4.82) for the highest quartile vs. lowest quartile). After adjusting for potential confounding factors, high pattern scores were associated with a high risk of colorectal cancer (OR (95% CI) = 9.98 (6.81–14.62) for the highest quartile vs. lowest quartile). When stratified by the *IL-17F* rs763780 genotype, this association was stronger for individuals carrying the C allele (*p* for interaction = 0.034), particularly for individuals with rectal cancer (*p* for interaction = 0.011). In conclusion, a dietary pattern reflecting inflammation was significantly associated with colorectal cancer risk. Moreover, this association could be modified according to the *IL-17F* rs763780 genotype and anatomic site.

## 1. Introduction

Chronic low-grade inflammation may play an important role in colorectal carcinogenesis [1]. Patients with inflammatory bowel diseases have an increased risk of developing colorectal cancer [2], whereas those using aspirin or other anti-inflammatory agents have a reduced risk [3]. Therefore, circulating inflammatory protein levels are frequently used as biomarkers of chronic inflammation and potential candidates to predict colorectal cancer [4]. 

Numerous studies have reported that diet may affect inflammation [5]. Due to the complex combination and synergistic effects of foods and nutrients, diet should be evaluated as a whole [5,6]. However, predefined diet quality scores are based on recommended diets and thus do not account for correlations between diet components [7]. On the other hand, dietary patterns derived using principal component analysis or factor analysis aim to explain the total variation in food intake. Therefore, derived dietary patterns may not be able to predict disease. Recently, several studies have developed inflammation-specific dietary patterns or scores [8,9]. To investigate the role of diet in disease, both prior information regarding disease pathways and culture-specific dietary habits should be considered. Reduced rank regression (RRR) is a useful method to establish specific dietary patterns by reflecting the link between diet and disease [10]. 

The complex interactions between an individual’s genetic background and dietary factors may help to explain the underlying mechanisms [11]. Recent studies have reported that IL-17F can induce the expression of various chemokines, cytokines, and adhesion molecules and is involved in inflammation-related cancers [12]. rs763780 (T7488C), which is a genetic variant at the start codon of the *IL-17F* gene, can lead to a His-to-Arg substitution at amino acid position 161 and thus inhibits the function of IL-17F. This polymorphism has been reported to contribute to an increased risk of several malignant tumors, including colorectal cancer [13].

Based on this information, we aimed to identify dietary patterns that reflected the dietary habits of Koreans and the pathways involved in colorectal carcinogenesis (i.e., inflammation). Then, we investigated whether the derived dietary pattern was associated with the risk of colorectal cancer. We also conducted stratified analyses to examine whether this association differed according to the anatomic site, presence of *IL-17F* rs763780, and other risk factors.

## 2. Materials and Methods

### 2.1. Study Population

The cases included in this study were patients newly diagnosed with colorectal cancer between August 2010 and August 2013 at the Center for Colorectal Cancer of the National Cancer Center, Korea. Of the 925 patients who agreed to participate in the study and completed the questionnaires, two patients were excluded due to implausible energy intakes. Therefore, 923 patients were included in the analysis. The controls were recruited between October 2007 and December 2014 from individuals visiting the Center for Cancer Prevention and Detection at the same hospital for a health check-up program provided by the National Health Insurance Cooperation; this program covers the entire Korean population. Of the 9157 healthy subjects who agreed to participate in the study and completed the questionnaires, 120 subjects were excluded due to the implausible energy intakes. Of the remaining 9037 individuals, two controls per case were randomly selected and frequency-matched by sex and five-year age group. Additionally, individuals with missing blood samples and high-sensitivity C-reactive protein (hs-CRP) concentrations above 20 mg/L were excluded to avoid confounding effects due to infection or medication use [14]. Therefore, 695 cases and 1846 controls were selected to analyze the association between dietary patterns and colorectal cancer risk. For the genetic association, individuals with missing genotyping data were excluded. Therefore, 694 colorectal cancer patients and 1402 healthy controls were included (Figure S1). 

All participants provided written informed consent, and the study protocol was approved by the Institutional Review Board of the National Cancer Center (IRB No. NCCNCS-10-350 and NCC2015-0202).

### 2.2. Data Collection

Information regarding the demographic and lifestyle risk factors (e.g., smoking, alcohol consumption, and regular exercise) of the participants was collected via in-person interviews or structured questionnaires during initial recruitment prior to the cancer diagnosis. Dietary intake was assessed using a validated 106-item semi-quantitative food frequency questionnaire (SQFFQ), which was developed specifically for Koreans and included commonly consumed food items. The validity and reproducibility of the questionnaire can be found elsewhere [15]. Each subject provided their average eating frequencies and typical portion sizes for the year preceding the interview. These values were converted to obtain daily nutrient intake values using a scale with nine frequency categories and three portion size categories.

### 2.3. Genotyping and Biomarker Measurement

The plasma hs-CRP concentrations were measured according to the manufacturer’s instructions. Plasma samples were aliquoted and stored at −80 °C. hs-CRP was analyzed using an ELISA kit (catalog No: E-EL-H5134) from Elabscience (Houston, TX, USA). 

*IL-17F* rs763780 (T7488C) was selected based on its association with inflammation and colorectal cancer [13,16,17]. Genomic DNA was extracted using the MagAttract DNA Blood M48 Kit (Qiagen, Hilden, Germany) and the BioRobot M48 automatic extraction equipment (Qiagen, Hilden, Germany) according to the manufacturer’s instructions. Genotyping was performed using the MassARRAY iPLEX Gold Assay (Agena Bioscience, San Diego, CA, USA). To control for the genotyping quality, we included duplicate samples for 3% of the subjects in our initial genotyping analysis; the rate of discordance was <1%. 

### 2.4. Statistical Analyses

The differences in the demographic and lifestyle factors between the cases and controls were analyzed using the χ^2^ test for categorical variables and Student’s t-test for continuous variables. The Wilcoxon test was used to compare the blood CRP levels between the cases and controls. 

To derive a dietary pattern predictive of colorectal cancer risk, we applied RRR using food groups as predictors and the plasma CRP concentration as a response [10]. RRR calculated the linear functions of the food group intakes (dietary patterns) that explained as much of the variation in the CRP concentration as possible; we defined this dietary pattern as “CRP-dietary pattern.” A more detailed description of the RRR, including the SAS code and its application in nutritional epidemiology, can be found elsewhere [10]. Because the CRP concentration distribution was skewed toward higher values, a log transformation was used. Food intake was categorized into 32 food groups that were energy-adjusted using the residual method [18]. The 32 predefined food groups were as follows: grains, tubers, noodles, rice cakes, bread/cake/pizza, sweets, snacks/cereals, legumes, tofu/soymilk, nuts, red meat, meat by-products, poultry, fish, bonefish, seafood/seashell oils, seafood products, seaweeds, salted fermented seafoods, eggs, milk/cheese, ice cream/yogurt, fruit, fruit products, vegetables, kimchi/pickled vegetables, mushrooms, oils, carbonated beverages, coffee, tea/beverages, and condiments/seasonings. 

We used an unconditional logistic regression model to investigate the associations of the CRP concentration and CRP-dietary pattern score with colorectal cancer risk. The subjects were divided into quartiles based on the distribution of the controls. The group in the lowest quartile was considered the reference group. A multivariable model was adjusted for age, sex, family history of colorectal cancer, regular exercise, education, and total caloric intake. The median intake of each quartile was used as a continuous variable to test for trends. Stratified analyses were performed according to the anatomic location and sex. A multinomial logistic regression model was used for the analyses stratified by anatomic location (colon and rectum). 

To investigate whether the association of the CRP-dietary pattern with colorectal cancer risk differed based on the *IL-17F* rs763780 genotype, we conducted stratified analyses. The CRP-dietary pattern scores were categorized into two groups (high/low) based on the median intake levels in the control group. The χ^2^ test was used to determine the Hardy-Weinberg equilibrium (HWE) of *IL-17F* rs763780 in the control group. We examined (i) the main effect of the CRP-dietary pattern in the strata defined by *IL-17F* rs763780 and (ii) the combined effect of both rs763780 and the CRP-dietary pattern. Interactions were assessed using the likelihood ratio test by comparing the model including the interaction term with the model that contained only the main effects. In addition, we conducted stratified analyses to evaluate whether this interaction differed according to other risk factors (e.g., age, sex, BMI, and regular exercise). 

All statistical analyses were performed using SAS 9.4 (SAS Institute Inc., Cary, NC, USA). A two-sided *p*-value less than 0.05 was considered significant. 

## 3. Results

### 3.1. General Characteristics of the Study Population

The distribution of the characteristics of the controls and cases is shown in Table 1. Compared with the controls, the colorectal cancer patients had a greater family history of colorectal cancer (*p* = 0.006) and a low education level (*p* < 0.001); they were also unlikely to perform regular exercise (*p* < 0.001) and had higher energy intakes (*p* < 0.001). However, no differences were observed for age, sex, BMI, smoking, or alcohol intake. The CRP level was significantly higher in the colorectal cancer patients than that in the control subjects (*p* < 0.001). 

### 3.2. Association between the CRP Concentration and Colorectal Cancer Risk

Higher CRP levels were associated with an increased risk of colorectal cancer (OR (95% CI) = 3.58 (2.65–4.82), for the highest quartile vs. lowest quartile). When stratified by anatomic site, this association was stronger for those with colon cancer (OR (95% CI) = 4.70 (3.15–7.03), for the highest quartile vs. lowest quartile) than for those with rectal cancer (OR (95% CI) = 2.58 (1.76–3.77), for the highest quartile vs. lowest quartile). In an analysis stratified by sex, a slightly stronger association was observed for women than that for men (Table 2). 

### 3.3. Correlation between the CRP-Dietary Pattern and Food Groups/Nutrients

Among the 32 food groups, we identified food groups that explained most of the variance in the CRP-dietary pattern score and presented only those with high factor loading (>ǀ0.15ǀ). In terms of the CRP-dietary pattern score, the intakes of some food groups (e.g., fruits, fruit products, and vegetables) were inversely associated with the CRP-dietary pattern score. In contrast, other food groups (e.g., grains) were positively associated with the CRP-dietary pattern score. Although salted fermented seafood and carbonated beverages showed higher factor loading, their intake amounts were quite low. Additionally, we examined the correlation between the blood CRP concentration and food groups. The blood CRP concentration was also correlated with food groups with higher factor loadings, with the exception of some food groups (e.g., bonefish). When we examined the correlation between the CRP concentration and the CRP-dietary pattern score, we found that the CRP-dietary pattern score was positively correlated with the CRP concentration (*r* = 0.22, *p* < 0.001) and explained 4.3% of the CRP variation (Table 3). 

When we derived the CRP-dietary pattern separately by sex, the proportion of the score variation was slightly different for some food groups. However, the major contributors were similar for both men and women (Table S1). 

We also examined the correlations between the CRP-dietary pattern scores and inflammation-related nutrients. Individuals with higher scores had lower fiber, vitamin D, riboflavin, folic acid, vitamin E, Fe, flavan-3-ol, flavone, and flavonol intake levels (Table S2). 

### 3.4. Association between the CRP-Dietary Pattern and Colorectal Cancer Risk

A higher dietary pattern score was associated with an increased risk of colorectal cancer (OR (95% CI) = 9.98 (6.81–13.62) for the highest quartile vs. lowest quartile). This association was slightly stronger for those with rectal cancer (OR (95% CI) = 11.69 (6.81–20.05) for the highest quartile vs. lowest quartile) than it was for those with colon cancer (OR (95% CI) = 8.87 (5.55–14.18) for the highest quartile vs. lowest quartile). When stratified by sex, this difference was observed only for women (Table 4). We also examined whether the general characteristics differed according to the dietary pattern score. Compared to the lowest group of dietary pattern scores, the highest groups were more likely to be men (*p* < 0.001), have lower education levels (*p* < 0.001), smoke cigarettes (*p* < 0.001), and drink alcohol (*p* < 0.001) and were less likely to exercise regularly (*p* < 0.001) (Table S3). 

### 3.5. Interaction between IL-17F rs763780 and the CRP Dietary Pattern Regarding Colorectal Cancer Risk

The association between the CRP-dietary pattern scores and colorectal cancer risk was examined according to the *IL-17F* rs763780 genotype. When stratified by the *IL-17F* rs763780 genotype, the association was stronger for individuals carrying a C allele (OR (95% CI) = 7.44 (4.27–12.96) for the highest quartile vs. lowest quartile) than it was for those carrying a T allele (OR (95% CI) = 4.18 (3.45–5.05) for the highest quartile vs. lowest quartile) (*p* for interaction = 0.035). When stratified by anatomical site, this interaction was observed only for those with rectal cancer (*p* for interaction = 0.011); individuals with a C allele had a greater risk of rectal cancer when they consumed a highly inflammatory diet (OR (95% CI) = 12.06 (5.14–28.32)) than when they consumed an anti-inflammatory diet (Table 5). When stratified by other risk factors, a stronger interaction was observed for women (*p* for interaction = 0.018) and those who did not exercise regularly (*p* for interaction = 0.003) (Table S4).

## 4. Discussion

In the present study, we found a strong significant association between the CRP-dietary pattern and the risk of colorectal cancer. We also found that this association could be modified according to the *IL-17F* rs763780 genotype, anatomic site, and other risk factors. 

The effect of diet on inflammation has received attention due to the role of inflammation in various chronic diseases. Many studies have reported that healthy dietary patterns, such as the Mediterranean diet and healthy eating index, have anti-inflammatory effects, but these dietary patterns do not focus on inflammation [19]. Recently, index-based or empirically derived dietary patterns have been widely used to investigate the effects of the whole diet on inflammation [6]. Shivappa et al. [8] developed a dietary inflammatory index using literature searches and a global database. However, this method is mostly based on nutrient intake and does not consider particular dietary habits for different geographical regions. Tabung et al. [9] and Kazula et al. [14] developed dietary scores that reflected inflammation in specific populations using food groups. These authors reported that the derived scores predicted the inflammatory statuses of individuals appropriately. In the present study, we obtained a dietary pattern using the RRR method with the CRP concentration as a response. Accordingly, a high score for the CRP-dietary pattern was significantly associated with an increased risk of colorectal cancer. This association was much stronger than the associations reported in the previous study conducted by Park et al. [20], which identified three dietary patterns using a principal component analysis with the same data set used for this study. Therefore, we assumed that the CRP-dietary pattern in this study could predict the risk of colorectal cancer successfully. Therefore, RRR is an appropriate method to establish dietary patterns associated with colorectal cancer development by reflecting individuals’ inflammation statuses [10,21]. 

Inflammation may be involved in colorectal carcinogenesis by inducing DNA damage, suppressing DNA repair, stimulating cell proliferation and angiogenesis, and inhibiting apoptosis [1]. In the present study, a high CRP-dietary pattern score corresponded with a high CRP concentration, which was reported to be associated with an increased risk of colorectal cancer [22]. A high CRP-dietary pattern score indicates a diet high in grains and low in fruit and vegetables, implying low levels of beneficial components (e.g., fiber, antioxidants, and flavonoids). Numerous studies have consistently reported that consuming dietary patterns rich in fruit and vegetables attenuates the inflammatory state [23]. Dietary fiber and flavonoids may activate antioxidants, inhibit eicosanoid-generating enzymes, and modulate the production of proinflammatory molecules and gene expression to attenuate inflammatory responses [24,25,26]. Therefore, a lack of these beneficial components may increase proinflammatory cytokine production and reduce anti-inflammatory cytokine production [27]. Additionally, components of grains have been reported to increase intestinal permeability and activate the immune system to increase inflammation [28]. Thus, the quantity and quality of diet components reducing inflammation have the potential to prevent colorectal cancer. 

The effects of diet on inflammation and carcinogenesis could differ according to genetic variations in inflammation-related genes. IL-17F, which is an important member of the IL-17 family, plays a critical role in regulating inflammatory reactions, because it induces the expression of various chemokines, cytokines, and adhesion molecules [29]. Genetic variation in *IL-17F* rs763780 (T7488C) leads to the conversion of His to Arg at amino acid 161, which may suppress the expression and activity of IL-17F [16]. Therefore, these genetic variations may contribute to inflammation and cancer susceptibility [13]. Studies have reported a significant positive association between the *IL-17F* rs763780 variant and the cancer risk, including colorectal cancer [13,17]. *IL-17F* rs763780 is also associated with an increased risk of the development of Crohn’s disease or ulcerative colitis development [30,31]. The present study found that a proinflammatory diet was more harmful to those with a C allele than to those carrying a T allele; thus, diet intervention is necessary for individuals with a variant allele. Furthermore, the interaction between a proinflammatory diet and the *IL-17F* rs763780 genotype in relation to colorectal cancer risk may support inflammation as an important pathway involved in colorectal carcinogenesis. 

The effects of inflammation-associated dietary patterns may be modified according to other risk factors and anatomic sites. First, women and overweight and inactive individuals are more likely to be affected by proinflammatory diets. In addition, different associations according to the *IL-17F* rs763780 genotype were stronger for women and for overweight and inactive individuals. Obesity is known to contribute to the formation of a proinflammatory environment, because adipocytes are a pro-inflammatory cytokine source [32]. Furthermore, adipose tissue and sex hormones in women may affect inflammation and thus can subsequently modify the effects of diet on the circulating levels of inflammatory biomarkers [33]. Inactivity is also reported to increase inflammation [34]. Therefore, it is assumed that a proinflammatory status may increase the risk of colorectal cancer, particularly for those with a variant allele. Because individuals consuming a highly inflammatory diet are more likely to have an unhealthy lifestyle, properly managing lifestyle habits can improve the effects of dietary intervention. Second, the role of diet in inflammation and colorectal carcinogenesis seems to differ according to the anatomic site. In the present study, the interaction for the rs763780 genetic variant was stronger for those with rectal cancer. Because the colon and rectum have different physiological functions, including the intestinal transit time, metabolic enzyme activity, and fecal composition, dietary factors and genetic variants that regulate inflammation may have different effects based on the anatomic location [35]. However, the evidence still remains limited, precluding hypotheses about the underlying mechanisms. Therefore, further studies are required to determine how inflammation and genetic variants contribute differently to carcinogenesis in the colon and rectum.

This study has several limitations that should be considered. First, a main limitation of the present study was potential recall and selection bias due to its case-control design. Although our cases were newly diagnosed colorectal cancer patients, we could not rule out the possibility that some patients might have changed their dietary habits when they experienced the first symptoms of colorectal cancer. In addition, selection bias may have occurred, because the controls recruited from health examinations may be more health conscious than the general populations. Second, in the present study, we examined the associations in a cross-sectional manner. Thus, the findings may be susceptible to reverse causality. However, many epidemiological studies have reported that dietary patterns remain stable over time [36]. Third, the sample size was relatively small for the analyses stratified by anatomic site and other risk factors. Therefore, these analyses may not have had sufficient power to detect small interaction effects. 

## 5. Conclusions

This study showed that a dietary pattern reflecting inflammation, which was characterized by low fruit and vegetable intake and high grain intake, was highly associated with an increased risk of colorectal cancer. The association between a proinflammatory diet and colorectal cancer risk could differ by the *IL-17F* genetic variant, anatomic site, and other risk factors. The findings from the present study support inflammation as an important pathway in colorectal carcinogenesis. Therefore, targeting the levels of inflammatory markers could be an effective strategy for colorectal cancer, particularly for high-risk individuals with genetic variants. 

## Figures and Tables

**Table 1:** General characteristics of the study subjects.

	Controls (*n* = 1846)	Cases (*n* = 695)	*p*-Value ^2^
Age (years), mean (SD)	56.1 (9.1)	56.4 (9.6)	0.44
Female, *n* (%)	596 (32.3)	222(31.9)	0.87
Family history of colorectal cancer (yes) ^1^, *n* (%)	99 (5.4)	58 (8.4)	0.006
BMI, *n* (%)			
<25 kg/m^2^	1225 (66.4)	476 (68.5)	0.31
≥25 kg/m^2^	621 (33.6)	219 (31.5)	
Educational level, *n* (%)			
<12 years	282 (15.6)	252 (36.3)	<0.001
≥12 years	1521 (84.4)	443 (63.7)	
Smoking status, *n* (%)			
Never	818 (44.3)	315 (45.3)	0.65
Ever	1028 (55.7)	380 (54.7))	
Alcohol consumption, *n* (%)			
Never	560 (30.3)	210 (30.2)	0.95
Ever	1286 (69.7)	485 (69.8)	
Regular exercise (yes), *n* (%)	1048 (58.2)	226 (32.5)	<0.001
Total caloric intake (kcal/day), mean (SD)	1689.9 (560.4)	2018.7 (529.7)	<0.001
CRP (ng/mL), median (IQR)	101.5 (48.3, 217.3)	212.6 (88.4, 614.7)	<0.001

CRP, C-reactive protein; IQR, interquartile range; SD, standard deviation; ^1^ First-degree relative; ^2^ *p*-values were calculated using the χ^2^ test for categorical variables, *t*-tests for continuous variables, and the Wilcoxon rank-sum test for inflammatory markers.

**Table 2:** Association between the CRP concentration and the risk of colorectal cancer stratified by sex and anatomic site.

	CRP Quartiles ^1^				*p* for Trend
	Q1	Q2	Q3	Q4	
**All**					
**Colorectal cancer**					
CRP (ng/mL), median	32	71	146	483	
No. controls/cases	461/80	462/124	461/150	462/341	
OR (95% CI) ^2^	1.0 (ref)	1.42 (1.02, 1.98)	1.68 (1.21, 2.32)	3.58 (2.65, 4.82)	<0.001
**Colon cancer**					
CRP (ng/mL), median	31	72	146	466	
No. controls/cases	461/35	462/60	461/71	462/183	
OR (95% CI) ^2^	1.0 (ref)	1.66 (1.05, 2.61)	1.91 (1.23, 2.97)	4.70 (3.15, 7.03)	<0.001
**Rectal cancer**					
CRP (ng/mL), median	32	70	146	434	
No. controls/cases	461/45	462/61	461/77	462/150	
OR (95% CI) ^2^	1.0 (ref)	1.19 (0.77, 1.82)	1.45 (0.96, 2.19)	2.58 (1.76, 3.77)	<0.001
**Men**					
**Colorectal cancer**					
CRP (ng/mL), median	35	79	153	533	
No. controls/cases	310/60	315/81	312/103	313/229	
OR (95% CI) ^2^	1.0 (ref)	1.15 (0.77, 1.70)	1.40 (0.95, 2.06)	2.97 (2.09, 4.22)	<0.001
**Colon cancer**					
CRP (ng/mL), median	34	79	152	518	
No. controls/cases	310/27	315/37	312/47	313/114	
OR (95% CI) ^2^	1.0 (ref)	1.21 (0.71, 2.07)	1.48 (0.88, 2.48)	3.51 (2.20, 5.59)	<0.001
**Rectal cancer**					
CRP (ng/mL), median	35	79	153	494	
No. controls/cases	310/33	315/43	312/55	313/110	
OR (95% CI) ^2^	1.0 (ref)	1.08 (0.65, 1.80)	1.32 (0.81, 2.15)	2.45 (1.56, 3.83)	<0.001
**Women**					
**Colorectal cancer**					
CRP (ng/mL), median	28	69	125	394	
No. controls/cases	149/27	149/42	149/49	149/104	
OR (95% CI) ^2^	1.0 (ref)	1.42 (0.79, 2.53)	1.64 (0.93, 2.90)	3.46 (2.04, 5.87)	<0.001
**Colon cancer**					
CRP (ng/mL), median	28	59	125	380	
No. controls/cases	149/13	149/19	149/25	149/67	
OR (95% CI) ^2^	1.0 (ref)	1.35 (0.62, 2.94)	1.73 (0.82, 3.66)	4.70 (2.38, 9.28)	<0.001
**Rectal cancer**					
CRP (ng/mL), median	28	58	124	332	
No. controls/cases	149/14	149/21	149/23	149/34	
OR (95% CI) ^2^	1.0 (ref)	1.37 (0.65, 2.90)	1.45 (0.69, 3.04)	2.09 (1.03, 4.24)	0.043

CI, confidence interval; CRP, C-reactive protein; OR, odds ratio; Q, quartile; ^1^ The subjects were divided into quartiles based on the CRP concentrations of the controls; ^2^ Adjusted for age, sex, total caloric intake, family history of colorectal cancer, physical activity, and education.

**Table 3:** Major food groups of the CRP-dietary pattern, and their correlations with CRP concentration and CRP-dietary pattern scores ^1^.

Food Group ^2^	Loading ^3^	% Score Variation ^4^	Spearman Correlation				CRP-DP Quartile	
			Food vs. CRP		Food vs. CRP-DP		Intake (g/day) ^5^	
			*r*	*p*-Value	*r*	*p*-Value	Q1	Q4
**Positive association**								
Grains	0.30	16.7	0.08	<0.001	0.43	<0.001	465	703
Salted fermented seafood	0.29	15.4	0.13	<0.001	0.34	<0.001	0.2	1.1
Carbonated beverages	0.27	13.3	0.07	<0.001	0.29	<0.001	0	0
Poultry	0.17	5.0	0.08	<0.001	0.22	<0.001	2.2	3.4
Seafood/Seashell	0.17	5.1	0.05	0.009	0.20	<0.001	9.4	12.9
Oils	0.16	4.5	0.06	0.004	0.22	<0.001	2.3	4.6
Noodles	0.15	4.3	0.06	0.001	0.22	<0.001	19.1	36.9
Sweets	0.15	4.0	0.05	0.018	0.18	<0.001	3.5	6.5
**Inverse associations**								
Fruit	−0.34	20.9	−0.12	<0.001	−0.43	<0.001	232	67
Bonefish	−0.27	13.4	−0.01	0.53	−0.20	<0.001	3.2	1.7
Fruit products	−0.26	12.1	−0.09	<0.001	−0.35	<0.001	44.1	12.0
Vegetables	−0.25	11.5	−0.07	<0.001	−0.35	<0.001	221	119
Milk/Cheese	−0.22	9.0	−0.06	0.001	−0.30	<0.001	67.6	10.7
Nuts	−0.22	8.6	−0.08	<0.001	−0.33	<0.001	5.0	1.1
Tubers	−0.19	6.4	−0.03	0.10	−0.20	<0.001	45.3	28.7
Tea/Beverages	−0.19	6.9	−0.02	0.33	−0.23	<0.001	49.9	14.3
Seaweeds	−0.18	5.8	−0.05	0.013	−0.23	<0.001	2.3	1.2
Condiments/Seasonings	−0.15	4.0	−0.03	0.13	−0.18	<0.001	18.2	13.2

CRP-DP, C-reactive protein dietary pattern; Q, quartiles; ^1^ CRP-dietary pattern scores were obtained by RRR using 32 food groups as predictors and CRP as a response. The Spearman correlation coefficient (*r*) between the CRP-dietary pattern and the CRP concentration was 0.22 (*p* < 0.001); ^2^ The food group intakes were energy-adjusted; ^3^ Factor loadings less than ǀ0.15ǀ are not presented in the table for simplicity; ^4^ Explained proportion of score variation; ^5^ Median.

**Table 4:** Association between the CRP-dietary pattern score and colorectal cancer risk.^1^

	CRP-Dietary Pattern Score Quartiles				*p* for Trend
	Q1	Q2	Q3	Q4	
All					
Colorectal cancer					
CRP (ng/mL), median	88	101	126	160	
No. controls/cases	461/51	462/95	462/185	461/364	
OR (95% CI) ^2^	1.0 (ref)	2.49 (1.66, 3.73)	5.14 (3.50, 7.57)	9.98 (6.81, 14.62)	<0.001
Colon cancer					
CRP (ng/mL), median	87	100	121	149	
No. controls/cases	461/30	462/50	462/96	461/173	
OR (95% CI) ^2^	1.0 (ref)	2.31 (1.40, 3.83)	4.83 (3.00, 7.76)	8.87 (5.55, 14.18)	<0.001
Rectal cancer					
CRP (ng/mL), median	85	97	119	138	
No. controls/cases	461/20	462/44	462/85	461/184	
OR (95% CI) ^2^	1.0 (ref)	2.85 (1.60, 5.08)	5.64 (3.25, 9.97)	11.69 (6.81, 20.05)	<0.001
Men					
Colorectal cancer					
CRP (ng/mL), median	97	108	139	167	
No. controls/cases	312/30	313/87	312/137	313/219	
OR (95% CI) ^2^	1.0 (ref)	2.83 (1.75, 4.59)	4.89 (3.07, 7.78)	7.53 (4.75, 11.92)	<0.001
Colon cancer					
CRP (ng/mL), median	95	106	122	156	
No. controls/cases	312/15	313/45	312/64	313/101	
OR (95% CI) ^2^	1.0 (ref)	2.97 (1.59, 5.58)	4.59 (2.50, 8.44)	7.30 (4.01, 13.27)	<0.001
Rectal cancer					
CRP (ng/mL), median	93	103	127	153	
No. controls/cases	312/15	313/40	312/69	313/117	
OR (95% CI) ^2^	1.0 (ref)	2.58 (1.35, 4.95)	4.96 (2.67, 9.23)	7.74 (4.22, 14.22)	<0.001
Women					
Colorectal cancer					
CRP (ng/mL), median	68	85	107	155	
No. controls/cases	149/17	149/31	149/54	149/120	
OR (95% CI) ^2^	1.0 (ref)	2.04 (1.02, 4.06)	3.54 (1.84, 6.80)	7.27 (3.86, 13.70)	<0.001
Colon cancer					
CRP (ng/mL), median	68	80	110	149	
No. controls/cases	149/11	149/16	149/32	149/65	
OR (95% CI) ^2^	1.0 (ref)	1.64 (0.70, 3.82)	3.33 (1.52, 7.28)	6.38 (2.99, 13.63)	<0.001
Rectal cancer					
CRP (ng/mL), median	66	85	98	121	
No. controls/cases	149/6	149/14	149/22	149/50	
OR (95% CI) ^2^	1.0 (ref)	2.53 (0.91, 7.00)	3.89 (1.46, 10.34)	7.99 (3.11, 20.52)	<0.001

CI, confidence interval; CRP, C-reactive protein; OR, odds ratio; Q, quartile; ^1^ CRP-dietary pattern scores were obtained by RRR using 32 food groups as predictors and CRP as a response; ^2^ Adjusted for age, sex, total caloric intake, family history of colorectal cancer, physical activity, and education.

**Table 5:** Association between the CRP-dietary pattern score and colorectal cancer risk stratified by the *IL-17F* rs763780 genotype.

	No. Controls/Cases		Combined Effect of the Dietary Pattern Score and rs763780		Effect of the Dietary Pattern Score by rs763780	*p* for Interaction
	Low DP Score	High DP Score	OR (95% CI) ^1^		OR (95% CI) ^1^	
			Low DP Score	High DP Score	High vs. Low	
Colorectal Cancer						
T allele	1217/255	1258/967	1.0 (ref)	3.87 (3.22, 4.66)	4.18 (3.45, 5.05)	0.035
C allele	183/27	146/139	0.75 (0.48, 1.16)	4.81 (3.52, 6.55)	7.44 (4.27, 12.96)	
Colon cancer						
T allele	1217/138	1258/477	1.0 (ref)	3.68 (2.93, 4.63)	4.17 (3.29, 5.30)	0.34
C allele	183/18	146/65	0.87 (0.51, 1.47)	4.24 (2.90, 6.20)	5.36 (2.78, 10.32)	
Rectal cancer						
T allele	1217/113	1258/473	1.0 (ref)	4.08 (3.18, 5.22)	4.14 (3.20, 5.35)	0.011
C allele	183/9	146/69	0.62 (0.32, 1.20)	5.31 (3.61, 7.80)	12.06 (5.14, 28.32)	

CI, confidence interval; CRP, C-reactive protein; DP, dietary pattern; OR, odds ratio. ^1^ Adjusted for age, sex, total caloric intake, family history of colorectal cancer, physical activity, and education.

## Supplementary Materials

The following are available online at http://www.mdpi.com/2072-6643/10/6/724/s1, Figure S1: Flow diagram of the cases and control selection, Table S1: Variations and factor loadings of the CRP-dietary pattern according to sex, Table S2: Correlation between the CRP-dietary pattern scores and the nutrient intake, Table S3. General characteristics of the study subjects according to the CRP-dietary pattern score quartiles, Table S4. Association of the CRP-dietary pattern score with the risk of colorectal cancer stratified by *IL-17F* rs763780 genetic variants and risk factors.
