# Supplementary material for: Inflammatory Dietary Pattern, IL-17F Genetic Variant, and the Risk of Colorectal Cancer
Source: Nutrients. 2018 Jun 5;10(6):724. doi: 10.3390/nu10060724 (PMC6024771; doi:10.3390/nu10060724)
Supplement: Supplementary file 1 [file nutrients-10-00724-s001.pdf]

Supplementary

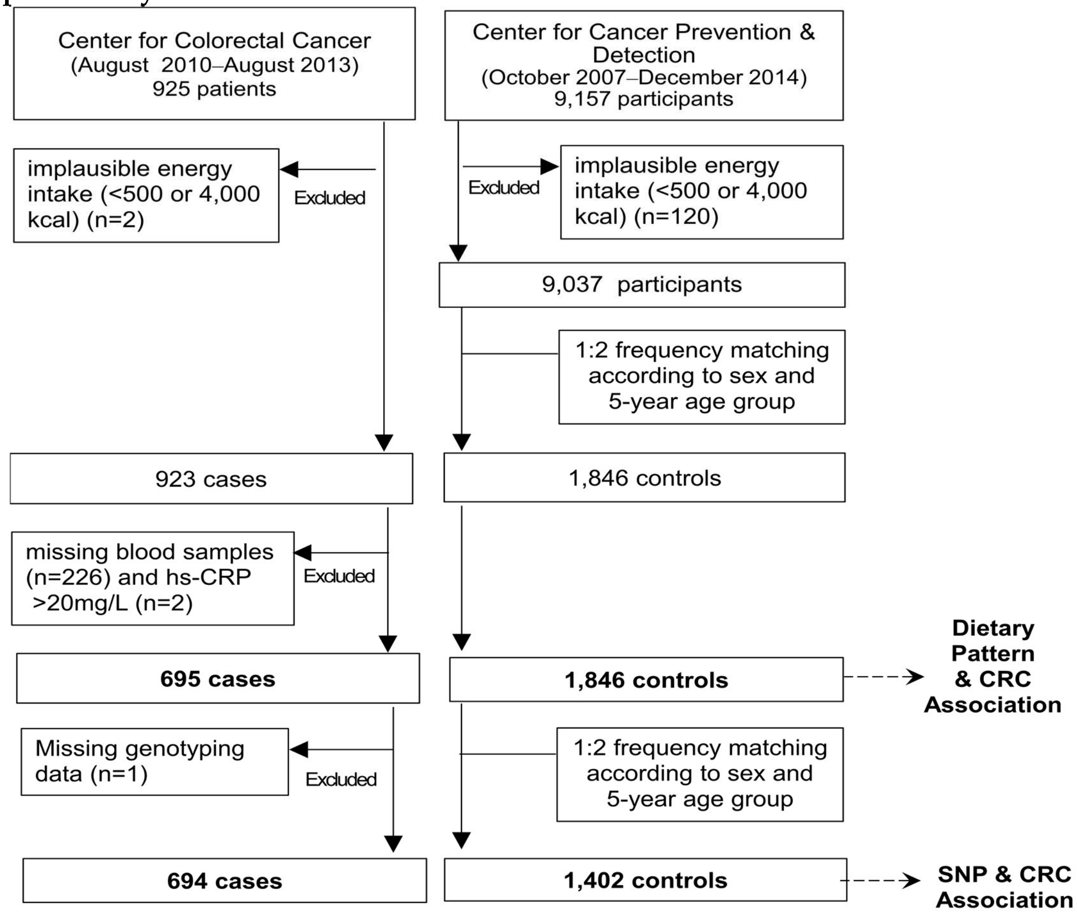

Figure S1. Flow diagram of the case and control selection.

Table S1. Variations and factor loadings of the CRP-dietary pattern according to sex.

Food group	Men		Women	
	Explained Proportion of Score Variation	Loading[1]	Explained Proportion of Score Variation	Loading[1]
Grains	3.75	0.16	21.31	0.40
Tubers	3.09	-0.15	2.96	-0.15
Noodles	7.10	0.22	-	-
Sweets	4.41	0.18	-	-
Tofu/Soymilk	6.20	-0.21	-	-
Nuts	4.83	-0.18	8.19	-0.25
Meat by-products	-	-	3.11	-0.15
Poultry	-	-	2.30	0.26
Bonefish	8.38	-0.24	8.11	-0.25
Seafoods/Seashells	3.36	0.15	6.87	0.23
Seaweeds	5.53	-0.20	-	-
Salted fermented seafoods	13.06	0.30	12.30	0.30
Milk/Cheese	3.46	-0.16	6.49	-0.22
Fruits	8.90	-0.25	10.10	-0.28
Fruit products	5.14	-0.19	5.09	-0.20
Vegetables	3.30	-0.15	13.19	-0.31
Oils	12.68	0.30	-	-
Carbonated beverages	26.09	0.43	-	-
Tea/beverages	5.98	-0.21	4.15	-0.18
Condiments/seasonings	-	-	4.70	-0.19

[1] Factor loadings less than |0.15| are not presented in the table for simplicity.

Table S2. Correlation between the CRP-dietary pattern scores and the nutrient intakes.

**Nutrients**	**Spearman Correlation Food vs. CRP-DP**		**Intake, Median**			
	r	***P*-Value**	**Q1**	**Q2**	**Q3**	**Q4**
Energy (kcal)	-	-	1733	1722	1732	1702
Protein (g)	-0.09	< 0.001	67.6	65.4	63.7	63.8
Fat (g)	-0.13	< 0.001	33.5	30.5	29.6	29.2
Carbohydrate (g)	0.12	< 0.001	333	341	344	346
Fiber (g)	-0.41	< 0.001	24.6	20.5	18.2	17.3
Monounsaturated fatty acids (g)	-0.05	0.012	8.7	8.1	7.7	8.0
Saturated fatty acids (g)	-0.07	0.001	8.3	7.7	7.3	7.5
Polyunsaturated fatty acids (g)	-0.07	< 0.001	5.3	5.0	4.8	4.9
n-3 fatty acids (g)	-	-	0.6	0.5	0.5	0.6
n-6 fatty acids (g)	-0.05	0.017	4.3	4.1	4.1	4.0
Cholesterol (mg)	-0.12	< 0.001	204	177	165	158
Thiamin (mg)	-0.03	< 0.001	1.1	1.0	0.9	0.9
Riboflavin (mg)	-0.37	< 0.001	1.1	0.9	0.9	0.8
Niacin (mg)	-0.21	< 0.001	14.9	13.6	13.0	12.7
Vitamin B6 (mg)	-0.33	< 0.001	1.7	1.5	1.4	1.4
Vitamin B12 (ug)	-0.11	< 0.001	9.0	7.7	7.0	7.4
Vitamin C (mg)	-0.46	< 0.001	141	103	86	75
Folic acid (ug)	-0.35	< 0.001	598	495	444	434
Vitamin A (ug RE)	-0.33	< 0.001	757	583	499	477
Vitamin D (ug)	-0.18	< 0.001	3.1	2.5	2.2	2.1
Vitamin E (mg)	-0.38	< 0.001	10.2	8.6	7.9	7.6
Beta-carotene (mg)	-0.29	< 0.001	4.1	3.0	2.6	2.5
Iron (mg)	-0.37	< 0.001	12.6	13.6	12.4	12.0
Magnesium (mg)	-0.13	< 0.001	128	115	113	115
Selenium (ug)	0.21	< 0.001	93.2	95.7	97.8	99.4
Zinc (mg)	-0.07	< 0.001	10.4	10.1	9.9	10.1
Ethanol (g)	0.29	< 0.001	5.5	8.1	12.2	19.9
Flavan-3-ols (mg)	-0.39	< 0.001	25.8	13.7	10.2	6.6
Flavones (mg)	-0.36	< 0.001	1.6	1.2	1.0	0.9
Flavonols (mg)	-0.38	< 0.001	26.5	19.0	15.5	14.3
Flavanones (mg)	-0.30	< 0.001	7.2	4.4	3.3	2.3
Anthocyanidins (mg)	-0.30	< 0.001	25.7	19.7	16.3	14.3
Isoflavones (mg)	-0.15	< 0.001	31.4	26.5	23.7	22.6

CRP-DP, C-reactive protein dietary pattern; Q, quartile

Table S3. General characteristics of the study subjects according to the CRP-dietary pattern score quartiles.[1]

	Dietary Pattern Score Quartile				*P*-Value[3]
	Q1	Q2	Q3	Q4	
Age (years), mean	56.4	55.7	55.9	56.5	0.50
Sex, n (%)					
Male	232 (13.5)	368 (21.4)	475 (27.6)	648 (37.6)	<0.001
Female	280 (34.2)	189 (23.1)	172 (21.0)	177 (21.6)	
Family history of colorectal cancer (yes) [2], n (%)					
No	476 (20.0)	529 (22.2)	609 (25.6)	766 (32.2)	0.21
Yes	36 (22.9)	26 (16.6)	36 (22.9)	59 (37.6)	
BMI, n (%)					
<25 kg/m^2	347 (20.4)	384 (22.6)	444 (26.1)	526 (30.9)	0.12
≥25 kg/m^2	162 (19.6)	173 (20.6)	203 (24.2)	299 (35.6)	
Educational level, n (%)					
< 12 years	71 (13.3)	81 (15.2)	131 (24.5)	251 (47.0)	<0.001
≥ 12 years	430 (21.9)	463 (23.6)	506 (25.8)	565 (28.8)	
Smoking status, n (%)					
Never	313 (27.6)	263 (23.2)	259 (22.9)	298 (26.3)	<0.001
Ever	199 (14.1)	294 (20.9)	388 (27.6)	527 (37.4)	
Alcohol consumption, n (%)					
Never	223 (29.0)	179 (23.3)	172 (26.6)	196 (25.5)	<0.001
Ever	289 (16.3)	378 (21.3)	475 (26.8)	629 (35.5)	
Total caloric intake (kcal/day), mean	1810.9	1803.1	1777.6	1746.4	0.57
Regular exercise, n (%)					
No	172 (14.1)	212 (17.4)	330 (27.0)	507 (41.5)	<0.001
Yes	335 (26.3)	330 (25.9)	304 (23.9)	305 (23.9)	
CRP (ng/mL), median	88.3	101.2	126.0	160.2	<0.001

BMI, body mass index; CI, confidence interval; CRP, C-reactive protein; OR, odds ratio; Q, quartiles; [1] CRP-dietary pattern scores were obtained by RRR using 32 food groups as predictors and CRP as a response. The subjects were divided into quartiles based on the CRP concentrations in the controls; [2] First-degree relative; [3] *P*-values were calculated using the χ^2 tests for categorical variable, t-tests for continuous variables, and Wilcoxon rank-sum tests for inflammatory markers.

Table S4. Association of the CRP-dietary pattern score with the risk of colorectal cancer, as stratified by the *IL-17F* rs763780 genetic variant and risk factors.[1]

IL-17F rs763780	T allele			C allele			*P* for Interaction
	No. Controls/Cases		High vs. Low	No. Controls/Cases		High vs. Low	
Pattern score	Low	High	OR (95% CI)[2]	Low	High	OR (95% CI)[2]	
**Age**							
< 50 years	240/89	308/186	1.84 (1.30, 2.61)	46/9	34/30	3.87 (1.46, 10.20)	0.08
≥ 50 years	977/166	950/781	5.78 (4.54, 7.23)	137/18	112/109	10.06 (4.98, 20.35)	0.18
**Sex**							
Men	693/127	1001/697	4.03 (3.16, 5.15)	105/15	119/105	5.60 (2.82, 11.16)	0.39
Women	524/128	257/270	4.27 (3.13, 5.82)	78/12	27/34	10.20 (4.05, 25.91)	0.018
**BMI**							
< 25 kg/m^2	839/170	801/665	4.81 (3.81, 6.09)	127/20	103/95	6.27 (3.28, 11.98)	0.27
≥ 25 kg/m^2	378/85	457/302	3.22 (2.31, 4.49)	56/7	43/44	12.40 (3.92, 39.25)	0.050
**Regular exercise**							
No	371/161	617/670	3.37 (2.61, 4.35)	69/13	71/92	10.37 (4.42, 24.36)	0.003
Yes	841/94	633/297	5.36 (4.01, 7.18)	113/14	75/47	5.56 (2.68, 11.71)	0.93

BMI, body mass index; CI, confidence interval; CRP, C-reactive protein; OR, odds ratio; [1] CRP-dietary pattern scores were obtained by reduced rank regression using CRP as a response variable. Subjects were divided into two groups based on the concentrations of CRP in controls; [2] Adjusted for age, sex, total caloric intake, family history of colorectal cancer, physical activity, and education, if applicable.
